# A Comprehensive Review on Bio-Based Materials for Chronic Diabetic Wounds

**DOI:** 10.3390/molecules28020604

**Published:** 2023-01-06

**Authors:** Jinjin Pei, Chella Perumal Palanisamy, Phaniendra Alugoju, Naga Venkata Anusha Anthikapalli, Prabhu Manickam Natarajan, Vidhya Rekha Umapathy, Bhuminathan Swamikannu, Selvaraj Jayaraman, Ponnulakshmi Rajagopal, Sirilux Poompradub

**Affiliations:** 1 Qinba State Key Laboratory of Biological Resources and Ecological Environment, 2011 QinLing-Bashan Mountains Bioresources Comprehensive Development C. I. C, Shaanxi Province Key Laboratory of Bioresources, College of Bioscience and Bioengineering, Shaanxi University of Technology, Hanzhong 723001, China; 2 Department of Chemical Technology, Faculty of Science, Chulalongkorn University, Bangkok 10330, Thailand; 3 Center of Excellence in Green Materials for Industrial Application, Faculty of Science, Chulalongkorn University, Bangkok 10330, Thailand; 4 Natural Products for Neuroprotection and Anti-Ageing Research Unit, Chulalongkorn University, Bangkok 10330, Thailand; 5 Department of Chemistry, A.N.R College, Gudivada, Andhra Pradesh 521301, India; 6 Department of Clinical Sciences, Center of Medical and Bio-allied Health Sciences and Research, Ajman University, Ajman 346, United Arab Emirates; 7 Department of Public Health Dentistry, Sree Balaji Dental College and Hospital, Pallikaranai, Chennai 600100, India; 8 Department of Prosthodontics, Sree Balaji Dental College and Hospital, Pallikaranai, Chennai 600100, India; 9 Centre of Molecular Medicine and Diagnostics (COMManD), Department of Biochemistry, Saveetha Dental College & Hospital, Saveetha Institute of Medical & Technical Sciences, Chennai 600077, India; 10 Central Research Laboratory, Meenakshi Academy of Higher Education and Research (Deemed to be University), Chennai 600078, India

**Keywords:** natural products, biomaterials, diabetic wounds, wound healing

## Abstract

Globally, millions of people suffer from poor wound healing, which is associated with higher mortality rates and higher healthcare costs. There are several factors that can complicate the healing process of wounds, including inadequate conditions for cell migration, proliferation, and angiogenesis, microbial infections, and prolonged inflammatory responses. Current therapeutic methods have not yet been able to resolve several primary problems; therefore, their effectiveness is limited. As a result of their remarkable properties, bio-based materials have been demonstrated to have a significant impact on wound healing in recent years. In the wound microenvironment, bio-based materials can stimulate numerous cellular and molecular processes that may enhance healing by inhibiting the growth of pathogens, preventing inflammation, and stimulating angiogenesis, potentially converting a non-healing environment to an appropriately healing one. The aim of this present review article is to provide an overview of the mechanisms underlying wound healing and its pathophysiology. The development of bio-based nanomaterials for chronic diabetic wounds as well as novel methodologies for stimulating wound healing mechanisms are also discussed.

## 1. Introduction

The skin barrier protects the body against many diseases and contaminants, including chemicals and microorganisms [1,2]. It consists of a variety of cells and tissues that are arranged in three layers: the epidermis located at the outer surface of the skin and the epithelium lining the skin, the dermis located in the middle, and the hypodermis located beneath [3]. Generally, surgery, burns, and chronic diseases can cause the skin to be damaged which makes it more likely to become infected [4]. A number of growth factors and inflammatory molecules interact to form new tissue during wound healing [5]. The replacement of damaged tissue by extracellular matrix (ECM) is also mediated by well-regulated protease activity. In order to heal damaged tissue and restore its integrity, it is important to design and develop new biocompatible and biodegradable materials [6]. The healing device should promote tissue regeneration, facilitate exudate clearance, and prevent infection at the site of the wound. Additionally, a local delivery of antibiotics at therapeutic doses can lessen wound microbial load, prevent infection spreading and systemic toxicities caused by oral administration [7]. The dressings might protect the wound environment from external contamination and contain substances that help wounds heal faster by inhibiting the growth of bacteria. There are several dressings that can be used to treat exudative wounds, but they often have inadequate fluid direction and absorption kinetics, which can cause tissue maceration and discomfort in the patient [8].

Nanotechnology-based self-healing processes similar to skin regeneration hold enormous promise [9]. A wide range of nanostructured materials have been developed for skin regeneration applications, including nanoparticles, spheres, capsules, emulsions, carriers, and colloids. In addition to providing mechanical strength and releasing chemical substances such as active drugs, antibiotics, growth factors, and other substances over a prolonged period of time, nanofibrous materials can also be used as scaffolds for skin regeneration [10]. Commonly, nanoparticles range in size from 1 to 100 nm, and they grow with serum and take on a pleomorphic shape [11]. The physical, chemical, and morphological properties of nanoparticles can be changed by changing their ion and protein compositions. A nanoparticle′s superior surface-to-volume ratio also makes it effective [12]. A few commercial products contain silver nanoparticles, as well as gold and gold nanoparticles in preclinical trials for wound healing. The silver nanoparticles stimulate epidermal re-epithelialization without forming scars by modulating inflammatory cytokines. The main mechanisms of silver nanoparticle toxicity have been identified as oxidative stress, Deoxyribonucleic acid (DNA) damage, and cytokine induction. The effects of silver nanoparticles on major organs have been measured by in vivo studies [13]. The consumption of gold nanoparticles inhibits the growth of bacteria, inhibits the formation of biofilms, and increases the proliferation and differentiation of keratinocytes [14]. Hydrogel dressings incorporating zinc oxide nanoparticles provide antibacterial properties for wound healing applications [15]. Also, metal nanoparticles are used as wound dressings and as delivery systems for antibacterial agents and other active drugs, along with polymeric nanoparticles such as chitosan, alginate, hyaluronic acid, polylactic acid (PLA), poly lactic-co-glycolic acid (PLGA), polycaprolactone (PCL), polyurethane, and cellulose [16]. In particular, alginate has high water absorption properties that keep wounds moist, while hyaluronic acid′s hygroscopicity facilitates cell adhesion, while hualuronan oligosaccharide polymers increase vascular endothelial growth factor (VEGF) expression, resulting in angiogenesis, proliferation, and migration of endothelial cells. A number of growth factors are also stimulated by cellulose, including VEGF, Basic fibroblast growth factor (bFGF), and platelet-derived growth factor (PDGF). As a result, wound healing is accelerated [17].

In order to speed wound healing, a variety of wound dressings have been developed, including semipermeable membranes, foams, hydrocolloids, and hydrogels. The fact that hydrogels are porous, hydrophilic, and biocompatible makes them a good choice for wound dressings, since they are highly hydrophilic, biocompatible, and resemble ECM in their three-dimensional (3D) structure. [18]. Several natural hydrophilic polymers (e.g., chitosan, gelatin, hyaluronic acid, alginate, and dextran) and synthetic hydrophilic polymers (e.g., poly(ethylene glycol) (PEG), poloxamer, poly (vinyl alcohol), olefin-containing polymerized monomers such as polyacrylamide (PAM), poly (acrylic acid), and polypeptides) have been used to create hydrogels through various chemical or physical crosslinks including dynamic chemical bonding, photo-crosslinking in situ polymerization, dual network, semi-interpenetrating network, and 3D printing [19]. Additionally, hydrogels are no longer limited to a single function or coverage but now combine multiple functions and show a trend towards further intelligence [20].

Aerogels, another significant biopolymer made from marine polymers such as chitosan and alginate, are desirable for use in wound healing [21]. Alginate provides moisture, and chitosan provides antimicrobial properties, in addition to the high porosity of the aerogel [22]. Chemical engineering, construction, and, more recently, the life sciences and medicine are just a few fields where aerogels are used. Biopolymers, in particular polysaccharides, have replaced inorganic materials since the first aerogels were made of inorganic elements such as silica. Using biopolymers of this class, aerogels can be made with enhanced characteristics such as biodegradability, which distinguish them from conventional ones. Marine polysaccharides, such as alginate and chitosan (deacetylated chitin), have become crucial in the synthesis of aerogels because of their natural availability as well as their physicochemical and biological properties [23].

A majority of the worldwide market for medical products is dominated by herbal remedies and plant-based medicines. Throughout history, herbal remedies and medications have played a crucial part in healing human illnesses. Although a wealth of literature is available on the curative properties of plant-based materials, there are currently no procedures for quality control regarding identification (phytochemical, pharmacological, and therapeutic activity) [24]. To ensure consistency and effectiveness, downstream processing involved in bioactive compounds from medicinal plants needs to be standardized. It is required to analyze herbal products′ medicinal effectiveness, using identification analysis, quality evaluation, and extract quality evaluation [25] in order to establish their legitimacy. Herbal treatments can help treat ulcers, heal wounds, and cure skin infections, scabies, leprosy, and venereal diseases due to their antiseptic properties. Plants are used in a variety of methods by folklore cultures to cure cuts, wounds, and burns including cleaning, debridement, and producing a moist environment conducive to natural healing [26]. Patents and articles indicate that herbal preparations are useful in treating wounds and accelerating wound healing. Several medicinal plants have demonstrated wound healing activity, including *Curcuma longa* (L.), *Terminalia arjuna*, *Centellaasiatica*, *Bidenspilosa*, *Aloe barbadensis*, and *Rauwolfia serpentine* [27].

## 2. Categorization of Wounds, Stages, and Impaired Wound Healing in Diabetes

**Figure 1:**
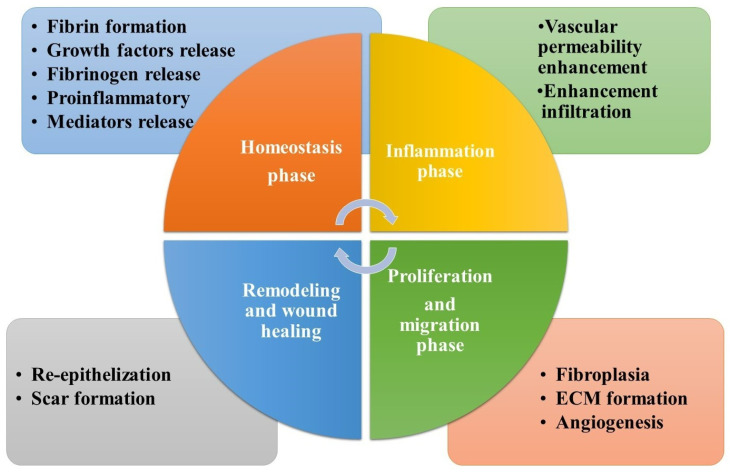
The stages of wound healing and the impact of biopolymers at each stage.

Wounds or injuries cause a person to lose fluids, suffer damage, and feel pain, as well as disrupt the skin′s structural integrity. [28]. Chronic wounds and acute wounds are two types of wounds. In chronic wounds, the healing process is complex and takes a long time, while acute wounds are those that heal quickly but cause significant agony to the patient. When a wound is chronic, it does not heal within a reasonable amount of time, even when treated with standard wound care methods and it does not heal within the regular timeframe of the healing process. Among several forms of chronic injuries are diabetic foot ulcers, burns, and ulcerative colitis. Depending on the size and depth of the lesion, acute wounds typically close within 8 to 12 weeks [29]. Inflammatory, hemostasis, and proliferative phases are the stages of healing (Figure 1) which last between one and four weeks, based on the scale and depth of the incision in the skin [30].

The blood clot is formed as soon as the hemostasis phase begins, when the exposed sub-endothelium, tissue factor, and collagen drive platelet aggregation, which induces degranulation and in turn the release of growth factors (GFs) and leads to the formation of a blood clot [31]. Neutrophils, proteases, and reactive oxygen species (ROS) all work together in the inflammatory phase to keep the wound free of microbial infection and clean it of debris, all in order to promote a speedier healing process. The redness, erythema, warmth, and swelling of wounded skin are all caused by exudate leucocytes [32]. To replenish the wound′s lost cells, the epithelial cells migrate in to replace them. The severity of the damage determines how long the hemostasis and inflammatory stages last [33].

In the proliferative phase, fibroblast cells multiply and connective tissue accumulates in profusion. ECM which includes hyaluronan (HA), proteoglycans, and additional species, produces granulation during the first clot formation [34]. The proliferative phase is supported by a wide range of cytokines and GFs. There are several members of this family, including members of the transforming growth factor-β family, angiogenesis factors (i.e., an epidermal GF), and the interleukin family [35]. This stage may last many days or even several weeks, depending on the person. Healing wounds reaches maturity when the wound has completely closed and has taken weeks or months to heal. After a month, the injured skin′s surface is totally covered in fibroblasts, which act as a new skin lining. As a new scar lining, fibroblasts cover the injury′s surface completely [36].

Diabetes is a long-term hyperglycemia-related metabolic condition. Uncontrolled blood glucose levels cause the bulk of diabetes-related problems, which are classified as either macrovascular or microvascular [37]. Diabetes affects approximately half a billion people worldwide, or over 10.5% of the adult population. Over 65-year-olds are most likely to develop type 2 diabetes. Over the next decade, it is also expected that type 2 diabetes will become more prevalent among the older population [38]. Type 2 diabetes is a serious illness that has become more common in recent years. To further simplify things, diabetes is further broken down into two primary categories: When it comes to Type-1 diabetes, the beta cells in the pancreas are destroyed by the immune system, which reduces insulin production. In contrast, Type-2 diabetes is caused by insulin resistance and a resulting decrease in insulin production. During pregnancy, gestational diabetes develops and it looks a lot like type 2 diabetes [39]. Other causes of diabetes include pancreatic illness, genetic beta-cell abnormalities, pollutants, and medicines. The healing time of wounds in diabetics is often longer than in healthy persons, which increases the risk of infection and other serious problems as well as raising healthcare costs [40].

Diabetes and wound healing factors: The healing process for diabetic wounds and other chronic infections differs from that for acute wounds, both experimentally and clinically. Several studies have addressed how wound healing might get stuck at a certain point in the process, which adds time to the healing process while also placing a significant financial and emotional load on the patient [41]. Vasculopathy: Diabetic patients with Type 1 Diabetes are more likely to have the macrovascular disease, and as a result, the distal arteries were less able to provide oxygen and essential minerals to a location of the wound as efficiently as those without the illness. It has been shown that diabetes changes blood vessel distribution in distal areas, including the metatarsal and femoral arteries and their branches [42]. When diabetes is first diagnosed, these microcirculatory abnormalities are common. As a result, the capillary′s diameter decreases, the basement membrane stiffens, and arteriolar deficits develop. As the breadth of the basement membrane expands, it inhibits physiological exchanges, reducing hyperemia and altering the body′s ability to regulate itself asymmetrically [43]. Recent studies in mouse models document deregulated angiogenesis and integrity of capillary beds as causative factors behind diabetic wound healing [44]. Endocrine dysfunction, such as the reduced activity of nitric oxide synthase, may develop in addition to the microcirculatory problems mentioned above. Blood flow can be irregular and wound healing can be subpar when there is endothelial cell dysfunction, which prevents the blood vessels from fully dilating [45].

Neuropathy: Because diabetes affects sensory, motor, and autonomic fibers, diabetic individuals have difficulty detecting external stimuli including pressure, heat, and wounds. The wound may not heal as quickly as it should as a result. Reduced ability to perceive pressure and reduced wound healing are both caused by a lack of pain and inconsistent vasodilator autoregulation. Damage to motor fibers and the aforementioned issues cause undue physical stress and wound aggravation. There is evidence to suggest that neuropathy has an involvement in the course of infection and microbial load in tissues. Infection: As a leading reason for death and hospitalization, infections also have devastating effects on a patient′s quality of life. When a diabetic sore or ulcer becomes infected, it can spread quickly. Cellulitis, osteomyelitis, and abscesses are serious medical conditions that need to be treated right away [46]. Even in the absence of infection-related symptoms such as fever or soreness, an elevated bacterial load is detrimental to wound, ulcer, and injury healing. Physiological events can be exacerbated by persistent hypoxia at the wound site, which increases the risk of reperfusion injury due to oxygen radical production [47].

The diabetic wound′s phases can be trapped for a longer period of time in either phase, and the model synchronization of the cascade disappears, which promotes rapid healing [48]. Early stages of healing following an injury include hemostasis and inflammation, during which inflammatory cells are drawn in by fibrin plugs being produced in the injured area [49]. The platelets release PDGF, transforming growth factor-1 (TGF-1), epidermal growth factor (EGF), and proinflammatory cytokines such as interleukin-1 (IL-1), to help neutrophils and macrophages bind to the injury site [50]. The decontamination of wounds by neutrophils is caused by the phagocytosis of bacteria at the wound site. It is believed that neutrophils and macrophages, as well as cytokines like interleukin (IL) 1 and 2, interleukin (IL) 6, interleukin (IL) 8, and tissue necrosis factor (TNF-1), all enhance inflammation by secreting growth factors such as EGF, TGF-1, PDGF, fibroblast growth factor, and IL-1 and IL-1. Chemotactic signals activate neutrophils, allowing the cells to be localized at the wound site. Once in the wound, neutrophils roll and transfer into the tissue, causing cell adhesion and ECM leakage. Diabetes-related microcirculatory abnormalities prolong the inflammatory phase and slow the healing process by preventing neutrophil adherence [51]. Growth factors are secreted, and tissue granulation is supported by fibroblasts and keratinocytes. Extracellular matrix and structural proteins are degraded by fibroblast metalloproteases (MMPs) that are produced by fibroblasts. To be sure, the extracellular matrix and damaged proteins are still present in diabetic wounds because the fibroblasts have grown resistant to endogenous growth hormones and cytokines [52].

The vascular system releases prostaglandin E2 (PGE2), which stimulates VEGF to promote angiogenesis and vasodilation. Low levels of PGE2 cause peripheral artery disease and vasculopathy because of high and/or variable glucose concentrations and limited oxygen availability [53]. Inflammatory vascular disease and impaired endothelial function cause reduced nitric oxide levels, increasing reactive oxygen species such as superoxide and hydrogen peroxide. This can exacerbate the wound. It is also detrimental to the healing of wounds, ulcers, and traumas when the bacterial load is elevated without the typical illness symptoms [54]. Enzymes such as hyaluronidase, elastase, and collagenase differentiate freshly recruited monocytes into macrophages by digesting hyaluronic acid, elastin, and collagen in connective tissue. Keratinocytes, fibroblasts, and endothelial cells are moved to the wound site during these procedures. [55]. Chronic inflammation is common in wounded tissues namely the feet and legs, ulcers, 2nd and 3rd degree burns, and diabetic wounds. These harmed tissues are made up of cells with insufficient oxygen uptake because of extracellular glycocalyx impairment [56]. Endothelial cell DNA damage happens when the cells are subjected to hypoxia and hyperglycemia, on the other hand. DNA damage can be prevented with the use of antioxidants like Silymarin. Injuries cause angiogenesis, which is the re-establishment of oxygen and nutrient supply, as well as an increase in growth factors and cytokines near the wounded area. Collagen, fibronectin, and connective-tissue growth factor (CTGF) are released by fibroblasts into the extracellular matrix. A unique type of fibroblast that aids in wound healing is the myofibroblast. It produces smooth muscle actin and focal adhesion protein. MMPs complete the healing process by remodeling the extracellular protein, which is formed and degraded by serine proteases [57]. Diabetic patients reportedly have four-stage wound healing that is slowed down. Vascular problems in diabetics are thought to be caused by endothelial dysfunction, malfunction of smooth muscle cells, and nerve-axon damage [58].

## 3. Biopolymer-Based Nanomaterials for Successful Wound Treatment

Organic compounds generated by living organisms are known as natural polymers or biopolymers [59]. An amino acid, monosaccharide, nucleoside, or ester can be made into a peptide, a polysaccharide, a polyphenol, and a polyester by covalently bonding these units together. Based on their properties, biopolymers from plants, animals, fungi, and bacteria can be used. Due to their biocompatibility, biodegradability, decreased antigenicity, and recyclability, these polymers outperform synthetics in many applications [60]. Due to their effective antibacterial, anti-inflammatory, and proliferative properties, numerous biopolymers, such as collagen, cellulose, chitosan, alginate, hyaluronan, and carrageenan, have been widely employed for wound healing (Table 1). These biopolymers have been developed into a new class of wound dressings using nanotechnology-based engineering methodologies [61]. Polymers derived from living organisms, such as bacteria, plants, and algae, have material properties that make it easy to mold them into hydrogels, scaffolds, and blends with other polymers to produce a skin substitute with enhanced mechanical strength, biomimetic characteristics, as well as other desirable characteristics [62].

**Figure 2:**
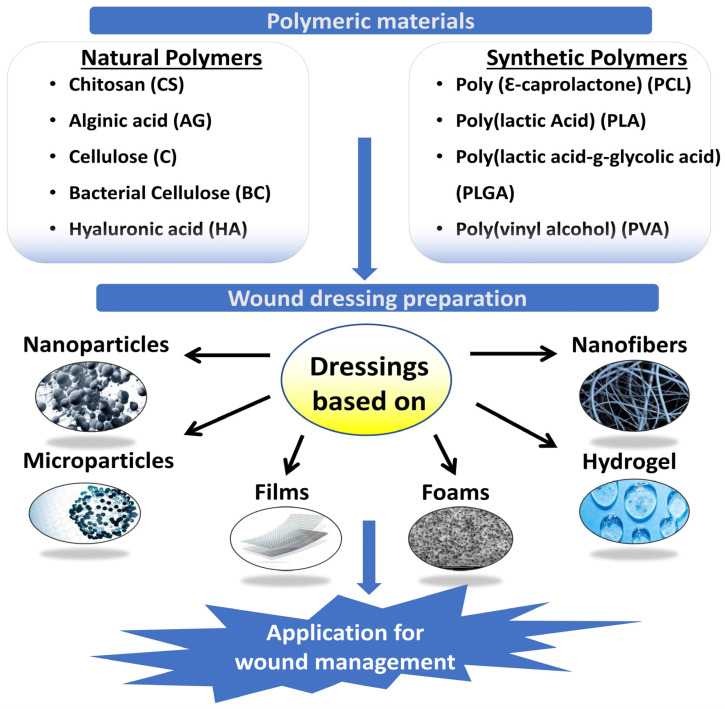
Polymeric materials for wound management.

Many biopolymers, which are polymers made by live microorganisms, are utilized in the treatment of wounds. Despite this, It looks as though some polymers receive increased awareness as wound dressings than others [63]. Wound dressings must be made to enhance and speed up the process of recovery. This can be done by shielding the wound from things like contamination and moisture loss, which could slow or impede healing [18]. Natural and synthetic polymers and their mixtures are employed in the wound dressings′ films, and sponges. Fibers or hydrogel-based hydrogels (Figure 2). In an ideal wound dressing, ECM of the skin would match the structure and biological features of the dressing. Due to their low cost, non-toxicity, and environmental friendliness, polymers found in nature are preferred for wound treatment over manufactured polymers [64]. Polysaccharides are natural polymers commonly used as wound dressings [65].

The morbidities associated with peritoneal adhesions after surgery include infertility, pain, and bowel obstruction. The researchers Ito et al. (2007) developed new injectable hydrogels based on dextran (DX) using hydrazide-modified carboxymethyldextran (CMDX–ADH) and aldehyde-modified DX (DX–CHO) or carboxymethylcellulose (CMC–CHO). A CMDX–DX gelling resulted in shrinkage, whereas a CMDX–CMC gelling resulted in swelling. Treatment of mesothelial cells and macrophages with CMDX-ADH and CMC-CHO, their cytotoxicity was minimal to mild, while that of DX-CHO was extremely high. The cytotoxicity of all cross-linked gels, however, was very mild. The CMDX–CMC formula significantly reduced the formation of adhesions in the rabbit sidewall defect-bowel abrasion model, while the CMDX–DX formula significantly worsened adhesion formation. Peritoneal adhesions were effectively prevented with cross-linked hydrogels of CMDX-ADH and CMC-CHO. There are potential advantages in preventing peritoneal adhesions with dextran-based hydrogels because they degrade slowly and are relatively inexpensive [66].

Chen et al. (2021) reported improved wound healing and anti-inflammation effectiveness by using GelMA/OD/Borax hydrogel as a hemostatic and anti-inflammatory hydrogel. GelMA was synthesized by modifying gelatin with methacrylic anhydride (MA) and polymerizing the polymer with UV light. Dextran′s o-hydroxyl groups are oxidized, producing aldehyde groups that bind with the -NH2 on tissue surfaces, forming the bonds that hold the tissues together. Additionally, the tri-network hydrogel showed excellent hemostatic capacity and was capable of withstanding high blood pressure of 165 mmHg, which exceeds the threshold for adults (120 mmHg). Hydrogels were effective in blocking bleeding even when used with Borax despite their mechanical properties, morphology, biocompatibility, and degradation. An innovative design modality has been used to develop a multifunctional hemostatizing hydrogel that can effectively stop bleeding and heal wounds [67].

Massive bleeding poses a threat to patient safety, which makes it a challenging clinical problem. In order to promote wound healing and repair, it is imperative to develop something with hemostatic and antibacterial capabilities. Xie et al. (2022) developed a multifunctional hydrogel containing carboxymethyl chitosan (CMCS)/sodium alginate (SA)/oxidized dextran (ODE) (CSO). In order to achieve fast hemostasis, the hydrogel used Schiff base and amide reactions in order to gel rapidly, adhere effectively to the wound site, and gel quickly. *Staphylococcus aureus* wound infections can be prevented with the hydrogel since CMCS and ODE possess antibacterial properties. A CSO hydrogel induced wound healing in mice by retaining water and mimicking the three-dimensional structure of the natural extracellular matrix. An in vitro whole-blood clotting assay demonstrated that red blood cells adhere well to hydrogel, resulting in good hemostasis after liver injury and tail amputation in rats. The biocompatibility of CSO hydrogel greatly improves hemostasis and wound healing [68].

An in situ cross-linking method was devised by Chen et al. (2022) to prepare carboxymethyl chitosan (CMCS)/oxidized dextran (OD)/polymeric glutamine acid hydrogels that promote hemostasis and antimicrobial activity. In the COP hydrogel, γ-PGA performed the function of draining the wound surface moisture and enhancing the adhesion with the surrounding tissues. Moreover, γ-PGA and CMCS can both electrostatically adsorb negative red blood cells and concentrate blood by absorption plasma. The antibacterial properties of CMCS and OD were imparted to COP hydrogel as a result of their antibacterial properties. The inhibition zone experiment demonstrated a clear boundary around the COP hydrogel. A study conducted in vivo demonstrated that COP hydrogel inhibited bacterial growth and promoted wound healing. A diffuse hemorrhage wound was hemostasized with COP hydrogel in a rat tail model. The multifunctional COP hydrogel is expected to be used in a variety of applications in order to achieve wound hemostasis and healing [69].

### 3.1. Collagen

Collagen is a common mammalian protein and an important part of ECM. Their triple helix structure is similar to that of collagen fibrils in terms of chemistry, with each fibril being a polymer made up of repeated amino acids linked together by peptides. The most prevalent collagen types in connective tissue are I, II, and III. In most conditions, collagen and proteolytic cleavage fragments, such as gelatin, help repair tissue due to their 29 different types [70].

Components such as type I collagen are essential for tissue healing. Numerous proteolytic enzymes are produced and cleave collagen into small parts during the inflammatory phase following damage. They are chemotactic to macrophages and mitogenic to fibroblasts, which leads to the production of granulation tissue and proliferation in the cells [71]. Arginylglycylaspartic acid (RGD) sequences are found in these fragments. A similar transition occurs between epithelium and mesenchyme in keratinocytes in the epithelium, facilitating tissue migration [72]. The delicate balance between collagen synthesis and breakdown determines whether or not a wound will heal properly. Because of the defective MMP/tissue inhibitors of metalloproteinase (TIMP) ratio, the newly generated collagens are split into fragments in chronic wounds, prolonging the inflammatory phase [73].

Increased surface modification, nanoscale reduction in collagen size, depolymerization, and labeling with anti-inflammation and anti-microbial chemicals can all increase the physiologic function of collagen [74]. Electrospinning, scaffold building, and combining biopolymers with medicinal compounds can improve their physical, mechanical, and functional properties. The ECM′s native collagen has closely resembled with electrospun collagen nanofibres coated with laminin which improves keratinocyte adhesion and migration [75]. Using silver oxide nanoparticles in methylcellulose hydrogels, Kim et al. (2018) developed a burn wound treatment option that enhances healing. By scavenging reactive oxygen species and promoting re-epithelialization, both quercitin and curcumin promoted diabetic wound healing in different experimental setups. In addition to sponges, hydrogels, films, membranes, powders, and freeze-dried sheets, collagen-based wound healing products are commercially available. Biopad, Helix bioactive collagen, and many other products fall into this category. It is important to choose the right product for the right wound type, as well as ensuring that the product contains the right active ingredients [76].

Indrakumar et al. (2022) synthesized the collagen scaffold encapsulating a metallic nanoparticle of molybdenum oxide (MoO_3_). Synthesized scaffolds were successfully fabricated, characterized, and biologically validated. Angiogenesis and wound healing were observed in the collagen sheet containing MoO_3_ nanoparticles. The use of trace elements in therapeutics is thus opened up by this study. This scaffold can be used to treat chronic wounds by further studying the roles of molybdenum in angiogenesis and cell proliferation pathways [77].

**Table 1:** Biopolymer characteristics and their biological role in the healing process.

Biopolymer	Monomer Units and Linkage	Sources	Biological Role	Advantages	References
Collagen	Amino acids connected through an amide bond	Bovine, porcine, etc.	It stimulates the production of ECM components by fibroblasts and acts as a chemotactic factor for macrophages	In order to reduce collagen surface size and polymerization, anti-inflammatory and antimicrobial chemicals can be added.	[78]
Cellulose	β-d-Glucose connected via the β-1,4-glycosidic bond	Plant cell wall and bacteria	Absorptive capacity of exudates, moisture retention	In tissue healing applications, cellulose-based biomaterials have been enhanced with biotechnological improvements to meet consumer requirements.	[79]
Alginic acid	α-1, 4 glycosidic links connect β-d-mannuronic acid and α-l-guluronic acid.	Brown algae	Proliferation and migration of fibroblasts are promoted by monocyte stimulation.	Numerous experiments have demonstrated that alginate is suitable for modification.	[80]
Hyaluronic acid	d-glucuronic acid and N-acetyl-d-glucosamine are glycosidically connected via β1,4 and β 1,3 glycosidic bonds.	Animal origin	Inhibits inflammation by increasing fibroblast proliferation and migration.	This substance accelerates wound healing by stimulating the proliferation of fibroblasts, remodeling of the extracellular matrix, and migration of keratinocytes. Hyaluronic acid breakdown products exhibit anti-angiogenic properties.	[81]
Chitosan	N-acetyl glucosamine is glycosidically connected via β-1, 4 glycosidic links.	Exoskeleton of crabs, molluscs, insects, fungal cell wall	Promotes the migration and proliferation of fibroblasts and keratinocytes	Chitosan has demonstrated promising wound healing results when combined with other biopolymers and surface modifications.	[82]

There has been an increase in the use of tissue engineering products as an alternative treatment for chronic wounds and burns. It has some disadvantages, such as additional steps and a lack of antibacterial characteristics, which might hinder wound healing; these problems must be adequately resolved for optimal wound healing [83]. Salleh et al. (2022) created a functionalized dual-layered hybrid biomatrix made of gelatin/cellulose hydrogel (outer layer) combined with graphene oxide and silver nanoparticles (GC-GO/AgNP) to fight potential post-implantation extraneous bacterial infection and collagen sponge (bottom layer) to promote cell proliferation and adhesion. GENIPIN 0.1% (*w*/*v*) was used to crosslink the bilayer hybrid scaffold for 6 h, followed by an advanced freeze-drying technique. Bilayer bioscaffolds were evaluated for their microstructure, biodegradability, surface wettability, antibacterial activity of nanoparticles, mechanical strength, and biocompatibility. Bilayer bioscaffolds showed good results for wound healing applications because their porosity enables them to absorb water and the biodegradation rate is slow. The biomatrix was also discovered to have hydrophobic qualities which are perfect for cell adhesion and mechanical strength. Additionally, using the disk diffusion technique, the hybrid GO-AgNP demonstrated antibacterial characteristics. Human dermal fibroblasts exhibited good cellular compatibility with the biomatrix. A potential application for the fabricated bilayer scaffold is the healing of skin wounds [84].

### 3.2. Cellulose

Most plant cell walls are made of cellulose, a biopolymer that is plentiful. d-glucose repeat units are bound together by 1,4-glycosidic connections in this compound. In contrast, bacterial cellulose is purer and more porous than plant cellulose. Certain bacteria from the Acetobacter, Sarcinaventriculi and Agrobacterium genera also generate it [85]. For the most part, the use of cellulose in wound management owes to its moisture-retentive qualities; moist injuries heal more quickly because growth factors and other healing-promoting chemicals are better supplied to the tissues. The porous cellulose structure resembles the skin′s ECM, assisting in tissue regeneration by aiding in the absorption of exudates (such as necrotic tissue and fibrinous covering) [86]. Improved functional qualities of cellulose have been achieved by bioengineering techniques such as surface immobilization with medicines or medicinal compounds, polymer blending, and electrospinning. Antimicrobials, such as those found in cellulose, can help keep the wound site free of infection [87]. Using myostatin, silver nanoparticles, and cellulose that has been impregnated with chloramphenicol, antibacterial activity against E. coli and Staphylococcus aureus has been documented [85]. Researchers evaluated cellulose-loaded therapeutic drugs such as vacarin, povidone iodide, and minocycline for their efficacy in drug release and wound healing qualities [88]. According to Lin et al. (2013) chitosan-coated bacteria-derived cellulose membranes improved epithelialization and regeneration more quickly than commercially available Tegaderm. Burn wound neovascularization and re-epithelialization were enhanced by UV-crosslinked cellulose and acrylic acid hydrogels. As a result of its nanoscale structure, nano-fibrillar cellulose has shown promising activity. Improved control of cellular responses has been demonstrated with cellulose functionalization through methylation and oxidation. Oxidized cellulose has also been found to exhibit hemostatic properties in research. Polyuronic acid is generated as a result of cellulose oxidation, and the resulting insoluble cellulose is known as oxidized cellulose. Compared to oxidized non-regenerated cellulose (ONRC), oxidized regenerated cellulose (ORC) has structured fibrils. Collagen and ORC alone, as well as when combined, both increase in vitro fibroblast proliferation [89].

In rabbits, carboxymethyl cellulose enhanced corneal epithelial repair, which was mediated by Zonula occludens (ZO)-1 expression, leading to a regeneration of the corneal epithelial barrier [90]. Human epidermal keratinocytes and fibroblasts were freeze-dried with bacterial cellulose and acrylic acid hydrogels to enhance recovery in incomplete thickness burn injuries. In animals with wounds, a 3D scaffold comprised of bacterial cellulose and gelatin exhibited full epithelial regeneration [91]. The healing potential of cellulose was examined at various pH levels. In a cutaneous wound model, acidic cellulose increased healing characteristics. Furthermore, polydopamine, tungsten oxide, and soy protein hydrolysate-conjugated cellulose enhanced integrin 1 expression, accelerated epithelialization, and exhibited antibacterial and anti-inflammatory activities [92]. In addition to retaining moisture, some cellulose-based dressings might act as a mechanical barrier and protect the delicate granulation tissue. Cellulose-based biomaterials have tremendous potential for tissue healing applications, and recent biotechnological advances have made it possible to enhance material properties to meet consumer needs [93].

### 3.3. Alginic Acid

The linear polymer of alginic acid is composed of repeated units of 1,4 glycosidic bonds between d-mannuronic acid and l-guluronic acid. Laminaria, Macrocystis, and Ascophyllum species generate alginic acid in maritime environments. By keeping the wound bed moist, absorbing excretions, reducing infection burden, removing odor, and aiding in hemostasis, alginates eliminate wound discomfort [94]. Alginate-containing dressings exchange calcium ions with sodium ions in blood or wound exudate when they come into contact with the exudate. The alginate fiber swells, partly disintegrates, and forms a gel after calcium ions are sufficiently supplanted by sodium ions. It has been shown that alginates stimulate monocytes to produce IL-6 and TNF-, which are essential for healing [95]. Alginate has poor cell adhesion capabilities on its own, but cell-interacting alginates can be synthesized by adding peptide sequences. A peptide sequence known as RGD is most commonly used for attachment, as it is recognized by integrin receptors. Alginates that resemble the structure of the ECM have been shown to increase cellular responses. When combined with several other polymers such as PVA and PCL, sodium alginate′s physical and mechanical properties have been demonstrated to be more stable. Alginate and PVA electrospun dressings have been shown to have superior properties to several commercial formulations [96].

Aloe vera extract combined with sodium alginate and cross-linked with UV light significantly decreased UV transmittance and shielded wounds from light [97]. Murakami et al. (2010) showed that a combination of chitosan, alginate, and fucoidan re-epithelialized a mitomycin-treated damaged wound [98]. Numerous such experiments demonstrate alginate′s suitability for modification. There have been several studies showing that alginate dressings improved healing, including those with curcumin or silver nanoparticles, silk fibroin composites and chitosan-alginate dressings. The freeze-dried composite of cellulose, chitosan, and alginate enhanced fibroblast and endothelial cell motility, acted as an anti-seawater barrier, and boosted EGF, bFGF, and CD31 expression in wounds with a full thickness [99]. Rats recovered from wounds faster when an EGF-loaded hydrogel was composed of N-carboxymethylchitosan and alginate. It was found that zinc crosslinked alginate showed enhanced epithelialization in rats compared with other divalent crosslinkers such as calcium, zinc, and copper [80]. Alginate is the active ingredient in a number of wound dressings, including 3M Tegaderm, Algicell, Algisite, Calcicare, Cutimed alginate, Dermalginate, Kaltostat, and Nu-derm. All of them are unique in terms of active substance composition and extra alterations made to fulfill the needs of particular wound types [100].

### 3.4. Hyaluronic Acid

Skin naturally contains glycosaminoglycans like hyaluronic acid. Evolution has conserved this biopolymer in mammalian cells. It consists of repeated units of N-acetyl-d-glucosamine and d-glucuronic acid that are linked by changing 1,4 and 1,3 glycosidic bonds. Hyaluronic acid is a chemical with a high molecular weight (5106 Da) [101]. Hyaluronan with a large molecular mass is anti-inflammatory, whereas fragmented hyaluronan has a pro-inflammatory effect. It is critical for wound healing because it stimulates fibroblast proliferation, ECM remodeling, and keratinocyte migration. It has been discovered that the breakdown product of hyaluronic acid has a pro-angiogenic action. Through binding to CD44 receptors on keratinocytes, it causes a cascade of events that promote differentiation [102]. Exogenous hyaluronic acid has been shown to decrease scar formation and collagen synthesis after injury even though hyaluronic acid is an endogenous biopolymer. The favorable properties of hyaluronan have made it an important therapy for wound healing [103]. Li et al. (2018) demonstrated that hyaluronic acid grafted with pullulan enhanced anti-enzymatic degradation. Additionally, it demonstrated faster healing due to its hemostatic activity. Furthermore, chitosan/hyaluronate hydrogels containing vancomycin microspheres increased endothelial cell proliferation at the wound site while reducing microbial burdens. When chitosan/polycaprolactone and hyaluronic acid scaffolds were electrospun with chitosan/polycaprolactone and aminoethyl methacrylate nano-hydrogels, the same effects were observed. Angiogenesis and regeneration of the dermis in diabetic ulcers were stimulated by cross-linking hyaluronan with epoxy and other layers. Applied to infected lesions, hyaluronic acid, gelatin, and chondroitin sulfate-based skin substitutes increased cell migration, reduced oxidative stress, and inhibited bacterial growth [104].

A photopolymerizedglycidyl methacrylate-hyaluronic acid compound exhibited enhanced hydrogel characteristics. Hu and colleagues demonstrated that lowering TGF-1 expression created a fetal-like environment for wound healing using a three-dimensional hyaluronic acid scaffold. It was discovered that applying topical hyaluronate to gingival surgery patients speeded up the healing process and reduced pain and swelling after the procedure. In rats, a mouldable hydrogel composed of hyaluronic acid, bisphosphonate, and silver demonstrated antibacterial and self-healing properties [105]. The chitosan/hyaluronate and edaravone conjugate demonstrated increased blood compatibility. Hyaluronic acid is found in commercial wound care products such as Dermaplex, Regenecare, Hyalomatrix, and Hyalofill, which provide cosmetic and protective benefits. [97].

### 3.5. Chitosan

Chitin, common in fungus, insects, molluscs, and crustaceans, consists of 1,4-linked N-acetylglucosamine molecules. In contrast to chitin itself, chitosan is an active deacetylated derivative. It has been demonstrated that chitosan encourages hemostasis and speeds up tissue regeneration [106]. Chitosan has been extensively explored as an antibacterial agent for wound infection prevention. Chitosan also promotes cell proliferation which is essential to healing, in addition to its antibacterial and antifungal properties. Polymorphonuclear leucocytes and macrophages are known to be stimulated by it for phagocytosis and the production of IL-1, TGF-, and PDGF [107]. Chitosan also promotes fibroblast proliferation, and fibroblast activation is positively correlated with the level of de-acetylation [108]. Hydrogels, foams, and scaffolds are made using chitosan and other polymers. When combined with other biopolymers and surface modifications, chitosan has shown promising wound healing results [109].

Using chitosan nanoparticles loaded into an Ag-metalorganic framework (0.1%Ag@MOF/1.5%CSNPs) and polyvinyl alcohol/sodium alginate/chitosan (PACS) as upper and lower layers, Zhang et al. (2021) successfully prepared a bilayer composite dressing for wound healing. Bilayer dressings were evaluated for their performance. PACS had good water retention, swelling, water vapor permeability, and biocompatibility, while PACS had barely any antibacterial activity. Antibacterial activity of the upper layer (Ag@MOF/CSNPs) was excellent, but biocompatibility was poor. In addition to preventing direct contact with the skin, it can also inhibit microbial growth. Moreover, the bilayer promotes blood coagulation and cell proliferation by adhering to a large number of red blood cells and platelets. CSNPs, Ag@MOF, Ag@MOF/CSNPs, and bilayer all displayed antibacterial activity in ascending order due to their synergistic antibacterial effects. A comparison of bilayer and PACS dressings in vivo revealed that the bilayer dressing accelerated wound healing significantly, whereas the PACS dressing showed less inflammation. Ultimately, this new bilayer composite dressing accelerates wound healing [110].

### 3.6. Patents on the Herbal Formulation for Wound Healing

Honey dressings were patented in 2015 by Sell et al. (Table 2) which are made from honey-impregnated alginate fiber sheets. Because of the porous surface of this dressing, it turns gel-like when applied to wounds after absorbing the exudate′s fluid. This patent contains eleven claims outlining the process through which honey is included into dressings. It is effective for both acute and chronic wounds [111]. Michael Koganov et al. (2013) patented “Bioactive compounds from theacea plants for wound and cut treatment” in 2013. It consists of a bioactive topical preparation containing bioactive components of theacea plants. An active component of theacea plants is capable of reducing inflammation and repairing skin or tissue damage [112]. Suresh Balkrishna et al. (2014) filed a patent for a “novel herbal formulation for wound healing.”. They have developed a regenerative medicine formulation that combines synergistic herbal components. The product is a combination of therapeutically effective extracts of *Curcuma longa*, *Glycyrrhiza glabara*, *Hamiltonia suaveolens*, *Tipha angustifolia* and *Azadirachta indica* as well as an optional base of Pig fat in *Sesamum indicum* (Til) oil [113].

Parveen Walia et al. (2014) patented a “Multifunctional natural wound healing matrix”. It contains a wound pad made of hydrophilic cotton fabric lined with organically manufactured silver nanoparticles and covered with zwitterionic low molecular weight chitosan. These ingredients also work together to boost therapeutic benefits with the addition of curcumin particles and tulsi extract which enhance their properties [114]. Additionally, Suresh Balkrishna et al. (2014) patented “A regenerative medicine, herbal composition for the treatment of wound healing.” Herbals that are typically used in wound and skin care formulations contain *Curcuma longa*, *Glycyrrhiza glabara*, *Hamiltonia suaveolens*, *Tipha angustifolia,* and *Azadirachta indica*. This features has 27 claims and six illustration sheets demonstrating testing on various wounds. The invention demonstrates the novel synergy and efficacy of herbal ingredients such as regenerative medication. Additionally, this demonstrates how the herbal composition is prepared [115].

**Table 2:** Patented technologies for wound healing based on medicinal plant constituents.

S.No	Patent No	Title	Remarks	Ref.
1	US 7,714,183 B2	Dressings made with honey	As a wound healing buffer, honey with the appropriate consistency and viscosity will be used in conjunction with a range of medical and surgical dressings.	[111]
2	US 2013/0146481 A1	Bioactive compositions from Theacea plants and processes for their production and use	In addition to protecting the skin tissue from UV-induced damage and normalizing skin issues, this treatment helps to prevent inflammatory behavior in mammals.	[112]
3	WO 2014/147638 Al	Wound healing matrix with several functions derived from nature	Cotton which is hydrophilic was used to synthesize the wound healing matrix in this study. Turmeric and tulsi extracts, as well as chitosan, were used to improve the efficacy of silver nanoparticles and were studied for their synergistic curative effects.	[114]
4	US 8,709,509 B2	A regenerative medicine based on a herbal composition for wound healing	A combination of herbal extracts of different plants (*Curcuma longa*, *Glycyrrhiza glabara*, *Hamiltonia suaveolens*, *Tipha angustifolia* and *Azadirachta indica)* in combination with Pig fat in *Sesamum indicum* (Til) were used for testing the synergistic therapeutic wound healing efficacy.	[115]
5	EP 2 896 396 A1	Herbal ointment for topical wound care	An herbal ointment/gel prepared from a combination of at least one of the pharmacological important plant extracts (Comfrey *Symphytum officinale* L. extract and/or *Commiphora molmol* tincture) and an antimicrobial agent (polyhexamethylenebiguanide and poloxameramer). Topical application of this herbal ointment is particularly useful for the treatment of skin and oromucosal wounds.	[112]
6	WO 2019/078931 Al	Dressing with buckwheat honey and bacitracin for wound healing	A mixture of buckwheat honey and bacitracin is applied directly for treating acute and chronic wounds and skin problems, as well as regenerating skin or dermal tissue in a chronic wound.	[112]
7	US 2019/0201474 A1	Herbal oil formulations for topical application and their therapeutic applications	This disclosure involves the use of Heterophragma roxburghii bark extract in the formulation of a herbal oil. This product is widely used in human and animal healthcare for treating wounds, skin conditions, and diseases linked to decreased blood flow.	[116]

During this period, some other researchers, Melikoglu et al. (2015) patented “Herbal formulation for topical wound dressing” using new herbal formulae which are effective for treating skin and oral mucosal wounds on a topical basis. In addition to polyhexamethylene biguanide and poloxamer as emulsifiers, a solution or gel that contains at least one medicinal component may increase analgesic, bacterial, antifungal, and antiinflammatory effects. (Comfrey *Symphytum officinale* L. extract and/or *Commiphora molmol* tincture). The preventive compound, algaecide, bactericide/bacteriostatic, fungicide/fungistatic, and microbicide/microbiostatic-polyhexamethylenebiguanide (PHMB) were utilized [112]. Kerri-Anne et al. patented a “Topical herbal formulation” that is especially effective for treating wounds and skin conditions. This mixture contains gotu kola (*Centella asiatica*), figwot (*Scrophularia nodosa*), yarrow (*Achillea millefolium*), *Plantago major* and *Echinacea purpurea*. Anti-inflammatory and antimicrobial characteristics are present in the formulation. It was discovered to be very beneficial as a combinatorial curing agent for wound therapy, scar prevention, and hair regeneration in the wound site. It was also proven to be effective in treating general skin diseases in humans, including as eczema and diaper rash [27].

Kenneth et al. (2017) created and patented a wound-healing bandage made of buckwheat honey and bacitracin. The innovation was shown to be beneficial in treating acute wounds and skin problems, as well as in regenerating skin and/or epidermal tissue in chronic wounds. The product is a blend of buckwheat honey and bacitracin in its composition or formulation. The composition is gelled in one distinct manifestation. An exuding or non-exuding acute or chronic wound or skin disease is treated by applying the mixture directly to the wound or by impregnating gauze or another similar product and applying it [117]. Dinesh et al. (2008) patented a “herbal oil mixture for topical use and medical purposes.” As part of the invention, a natural oil solution derived from *Heterophragma roxburghii* bark extract is developed that can be applied topically for treating and managing wounds and other medical problems associated with decreased blood flow in humans and animals, as well as a variety of skin irregularities and infections. Many medical conditions can be treated with the topical herbal oil formulation, including diabetic gangrene, dry gangrene, wet gangrene, athlete′s foot, burn wounds, diabetic foot ulcers, bedsores, untreated open sores, snakebite wounds, and cellulite-induced gangrene [116].

Tomulewicz et al. (2019) patented the “Herbal preparation for expediting the healing of wounds and skin inflammations and its application.” This invention relates to the treatment of wounds and skin inflammation with pharmaceutical preparations. This herbal medicine contains emulsified or suspended organic medium extracts of *Melittis melissophyllum* L. The different components in the herbal medicine are: An ointment was made with a concentration of 40% to 70% weight of Vaseline album and a 10% to 20% weight of ethyl alcohol. Triethylamine was used at a concentration of 2% weight-per-employer, hydroxy cellulose at 1% weight-per-employer, and filtered water, Aqua purificata, was used at a concentration of 30% to 35% weight-per-employer [118].

## 4. Future Perspective

**Figure 3:**
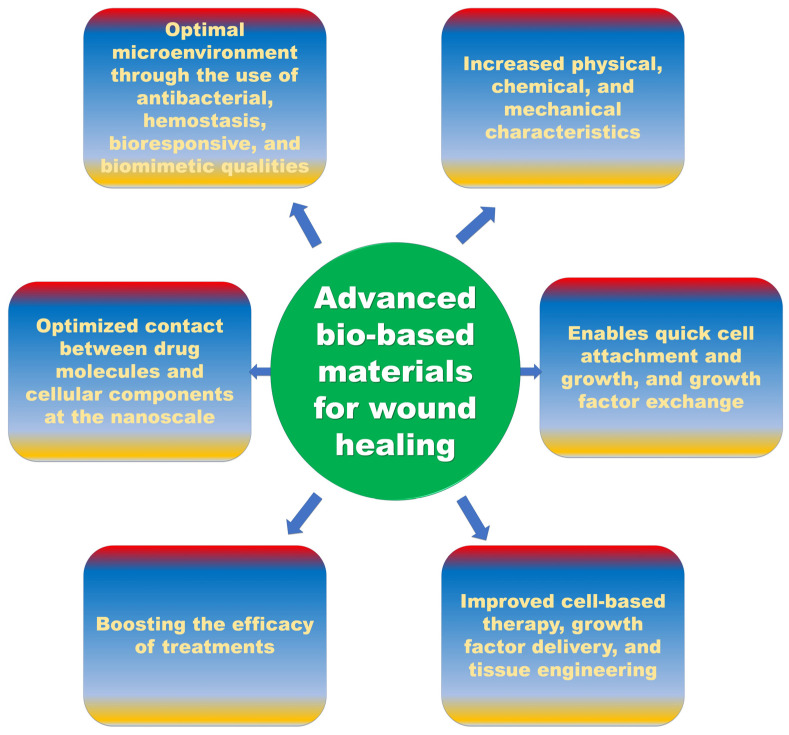
Future perspectives of bio-based materials for wound healing.

Despite the existence of various pre-existing materials, the outcomes of healing in chronic conditions remain hampered. Future wound care products (Figure 3) must inevitably perform several roles and create an optimal microenvironment through the use of antibacterial, hemostasis, bio responsive, and biomimetic qualities. The development of biopolymers will be aided by increased knowledge of their physical, chemical, and mechanical properties. Advances in regenerative medicine, nanotechnology, and bioengineering have made it possible to create scaffolds made from biopolymers, artificial skin models, and dressings infused with growth factors and drugs. Advances in nanotechnology and bioengineering technologies such as bioprinting and 3D electrospinning enable the creation of 3D scaffolds or matrices with chosen pore size and tensile strength that imitate the integrity of the skin. This enables quick cell attachment and cell growth, as well as appropriate nutrition and growth factor exchange. The contact between drug molecules and cellular components can be optimized at the nanoscale, boosting the efficacy of treatments. Since cell-based therapy, growth factor delivery, and tissue engineering are all being improved, we now have a solid platform for developing a substitute for the ECM that can mend damaged tissue.

## 5. Conclusions

Due to the technological advancements of modern society, nanomaterials are increasingly used in the treatment of wounds. Recent advances in bio-based materials that facilitate wound healing as well as the mechanisms that underlie them, are discussed in this paper. While there is a large body of literature on hemostasis, anti-infection, immunoregulation, and proliferation, further research is necessary to determine the appropriate mechanisms and the mechanisms of post-wound healing. As a result of their unique physicochemical and biological properties, nanoparticles have been found to be potentially useful for the delivery and release of therapeutic agents in wound dressings in a sustainable manner. Furthermore, animal models may be used to study the behavior of bio-based materials used in wound healing. Due to the differences between human and animal models, it is essential to find an alternative solution for preclinical studies. As a result, bio-based materials can be used in the future to improve the detection and treatment of wound infections.

## Data Availability

Not applicable.
